# The role of the desmosomal protein desmocollin 2 in tumour progression in triple negative breast cancer patients

**DOI:** 10.1186/s12935-023-02896-9

**Published:** 2023-03-16

**Authors:** Francesca Reimer, Sarah Bryan, Karen Legler, Thomas Karn, Serenella Eppenberger-Castori, Jakob Matschke, Thais Pereira-Veiga, Harriet Wikman, Isabell Witzel, Volkmar Müller, Barbara Schmalfeldt, Karin Milde-Langosch, Udo Schumacher, Christine Stürken, Leticia Oliveira-Ferrer

**Affiliations:** 1 Department of Gynaecology, University Medical Center Hamburg-Eppendorf, Martinistraße 52, 20246 Hamburg, Germany; 2 Goethe University, Frankfurt, Germany; 3 Institute for Pathology, Department of Molecular Pathology, University of Basel, Basel, Switzerland; 4 Institute of Neuropathology, University Medical Center Hamburg-Eppendorf, Hamburg, Germany; 5 Institute of Tumor Biology, University Medical Center Hamburg-Eppendorf, Hamburg, Germany; 6 Institute of Experimental Anatomy, University Medical Center Hamburg-Eppendorf, Hamburg, Germany; 7 MSH Medical School of Hamburg, University of Applied Sciences and Medical University, Hamburg, Germany

**Keywords:** DSC2, Desmosome, CTC cluster, Breast cancer, Brain metastasis, Pulmonary metastasis

## Abstract

**Background:**

The disruption of epithelial features represents a critical step during breast cancer spread. In this context, the dysregulation of desmosomal proteins has been associated with malignant progression and metastasis formation. Curiously, both tumour suppressive and pro-metastatic roles have been attributed to desmosomal structures in different cancer entities. In the present study, we describe the pro-metastatic role of the desmosomal protein desmocollin 2 (DSC2) in breast cancer.

**Methods:**

We analysed the prognostic role of DSC2 at mRNA and protein level using microarray data, western blot analysis and immunohistochemistry. Functional consequences of DSC2 overexpression and DSC2 knock down were investigated in the triple negative breast cancer (TNBC) cell line MDA-MB-231 and its brain-seeking subline MDA-MB-231-BR, respectively in vitro and in vivo.

**Results:**

We found a significantly higher DSC2 expression in the more aggressive molecular subtypes HER2-positive and TNBC than in luminal breast cancers, as well as a significant correlation between increased DSC2 expression and a shorter disease-free—also in multivariate analysis—and overall survival. Additionally, a significant association between DSC2 expression in the primary tumour and an increased frequency of cerebral and lung metastasis could be observed. In vitro, ectopic DSC2 expression or DSC2 down-regulation in MDA-MB-231 and MDA-MB-231-BR led to a significant tumour cell aggregation increase and decrease, respectively. Furthermore, tumour cells displaying higher DSC2 levels showed increased chemoresistance in 3D structures, but not 2D monolayer structures, suggesting the importance of cell aggregation as a means for reduced drug diffusion. In an in vivo brain dissemination xenograft mouse model, reduced expression of DSC2 in the brain-seeking TNBC cells led to a decreased amount of circulating tumour cells/clusters and, in turn, to fewer and smaller brain metastatic lesions.

**Conclusion:**

We conclude that high DSC2 expression in primary TNBC is associated with a poorer prognosis, firstly by increasing tumour cell aggregation, secondly by reducing the diffusion and effectiveness of chemotherapeutic agents, and, lastly, by promoting the circulation and survival of tumour cell clusters, each of which facilitates distant organ colonisation.

**Supplementary Information:**

The online version contains supplementary material available at 10.1186/s12935-023-02896-9.

## Background

Breast cancer is the most common malignancy among women and the leading cause of cancer-related death in women globally, with more than 90% of mortalities associated with stage IV metastatic breast cancer [1]. Breast cancer commonly metastasises to bone marrow, lung, liver, lymph nodes and brain tissue [2]. Four molecular subtypes, namely luminal A, luminal B, HER2-positive and triple-negative breast cancer (TNBC), have been associated with different patterns of metastatic spread; bone metastases with luminal A and B subtypes, liver metastases with HER2-positive subtype, and brain and lung metastases with TNBC and HER2-positive subtypes [3–5].

Metastases develop when malignant cells lose their connection to the primary tumour (dissemination), becoming circulating tumour cells (CTCs) which can be transported in blood to distant regions of the body [6–8]. Alongside single CTCs, circulating tumour cell clusters (CTC clusters) have been detected in the blood of cancer patients [9–13]. CTC clusters, which are disseminated cell aggregates of up to 50 tumour cells, have been shown to have an up to 50-fold higher chance of forming metastases than single CTCs, and are therefore associated with worse clinical outcomes [9, 14–16]. This higher metastatic potential may be attributed to the increased resilience of CTC clusters in circulation, compared to single CTCs, which in turn increases the probability of dissemination into a distant organ [12, 17]. Further, it has been shown that CTC clusters originate as cell aggregates from the original primary tumour, rather than through intravascular aggregation or proliferation of singular CTCs [9, 18]. However, the cellular and micro-environmental factors which promote CTC cluster formation are still largely unknown.

Desmosomal proteins have been previously described to be associated with cell aggregation and, in particular, high plakoglobin expression levels have been found in CTC clusters [9, 19, 20]. Desmosomes are cell junctions which stabilise the connection between neighbouring epithelial cells. In desmosomes, the transmembrane proteins desmocollin 1–3 and desmoglein 1–4, which belong to the cadherin superfamily, form homo- and heterophilic interactions and are intracellularly connected to the intermediate filament cytoskeleton through desmosomal plaque proteins, such as plakoglobin and plakophilin (members of the armadillo family) and desmoplakin (member of the plakin family of cytolinkers) [21–23]. One theory is that an abundant expression of desmosomal proteins could lead to enhanced intercellular adhesion between disseminating tumour cells, both during CTC formation and vascular transport [9, 18, 24]. To date, studies focusing on the expression of various desmosomal proteins in a variety of primary cancer types have shown both tumour-suppressive and tumour-promoting effects on growth and metastases [9, 25–27]. Recently, the desmosomal protein desmoglein 2 (DSG2) has been associated with a poorer prognosis and higher recurrence risk in breast cancer patients. DSG2 expression is regulated by hypoxia in breast cancer cells and increases the prevalence of CTC clusters, facilitating distant metastasis [25].

This study focused specifically on desmocollin 2 (DSC2)—a transmembrane cell anchoring protein—in primary breast cancer, for which the role in breast cancer metastases formation is still not completely understood. Several studies have found that DSC2 proteins are abnormally expressed in various types of cancer and correlate with cell proliferation and invasive behaviour [28, 29], and showed that a high expression of DSC2 increased cell aggregation [20, 30]. A recently published study by Li et al. accentuates that elevated DSC2 expression, in combination with the desmosomal protein Plakophilin-1 (PKP1), can activate PI3K/AKT or CDH1 to increase cluster formation to resist shear-stress-induced cell death. Furthermore, higher expression of DSC2 and PKP1 was correlated with lower overall survival and worse disease progression in patients with breast and lung cancer [31]. The aim of this study was to investigate the potential of DSC2 at mRNA and protein level as a predisposing factor for breast cancer progression and the development of breast cancer metastases, in particular to the lung and brain.

## Methods

### Patient cohorts

All patients, from whom the tissue samples were derived, were treated at the University Medical Centre Hamburg-Eppendorf, Germany, Department of Gynaecology between 1991 and 2002. All patients gave written approval for the utilisation of their tissue samples and the reviewing of their medical records according to our investigational review board and ethics committee guidelines (Ethik-Kommission der Ärztekammer Hamburg, #OB/V/03). Further cohort details and patient characteristics are listed in the Additional file 2: Table S1. Microarray analyses of DSC2 mRNA levels in patients with and without distant metastases were performed on 197 mRNA extracts from primary breast cancer tissue samples. For the western blot, a total of 111 samples were collected based on tissue availability from the same patient cohort. Slides of four tissue microarrays, constructed under permission of the Ethikkommission Beider Basel (EKBB # 395/11), kindly provided within collaborative efforts in the frame of the Pathobiology study group of the EORTC by Dr. Serenella Eppenberger-Castori from the Biobank at Institute of Medical Genetics and Pathology at the University of Basel, Switzerland, were used for immunhistochemical analysis. Patient characteristics are supplied in Additional file 3: Table S2.

### Microarray data

We analysed DSC2 mRNA levels using microarray data (Affymetrix HG-U133A) from the aforementioned cohort. Here, two probe sets (204750_s_at and 204751_x_at) corresponding to DSC2 were available and analysed independently. Additionally, the mean expression value of the 2 probe sets was calculated and also included in further analyses. According to the DSC2 mRNA values of each probe set and the mean value, the cohort was divided into quartiles of similar size, representing low, moderate-low, moderate-high, and high DSC2 levels. Correlations between DSC2 mRNA levels (quartiles) and clinicopathological factors, such as histological grading, stage, lymph node involvement, oestrogen, and progesterone receptor status (ER, PR) were statistically examined by χ^2^-tests. Overall survival was analysed by Kaplan–Meier analysis and log-rank tests. Additionally, the correlation between DSC2 mRNA levels (continuous data) and disease-free and overall survival was calculated using Cox regression analyses. Multivariate Cox regression analyses including the clinical stage, nodal involvement and molecular subtype were performed for all probe sets and the DSC2 mean value. Here, a backwards analysis with stepwise removal of insignificant terms was used. Probability values less than 0.05 were regarded as statistically significant. All statistical analyses were conducted using SPSS software Version 26 (SPSS Inc., Chicago, IL, USA). For validation purpose we used an independent Affymetrix microarray dataset consisting of 572 breast cancer samples from Gene Expression Omnibus (GSE2603, GSE2034, GSE12276) for which detailed information on metastatic localization was available [32].

### Protein lysate preparation and western blot analysis

Tissue samples were obtained intraoperatively and immediately stored in liquid nitrogen as fresh frozen samples. The histological characteristics of each sample were evaluated on cryo-cut and haematoxylin–eosin-stained sections. The tissue was tailored, where necessary, to obtain at least 70% tumour cells in the sample used for protein extraction. Approximately 100 mg of tissue was excised and pulverised using a micro-dismembrator (Braun-Melsungen, Melsungen, Germany) for 2 min and 45 s at 200 r.p.m. Proteins were lysed in ice-cold sample buffer (50 mM Tris pH 6.8, 1% sodium dodecyl sulphate (SDS)), 10% sucrose and 10 μl/ml protease inhibitor cocktail (Sigma, Taufkirchen, Germany). For western blot analyses, volumes of tumour lysates containing 20 μg of protein were loaded per well. The following antibodies were utilised in the western blot detection process: mouse monoclonal anti-DSC2 IgG (Millipore, MABT411) dilution 1:1000, mouse monoclonal anti-β-Actin (C4) (Santa Cruz Biotechnology, sc-47778) dilution 1:2000 and goat anti-mouse IgG-HRP (Santa Cruz Biotechnology, sc-2055) dilution 1:8000. Antibodies were visualised using a chemiluminescent reagent (SuperSignal ® West Pico chemiluminescent Substrate, Thermo Scientific, Rockford, USA). Protein band intensities were quantified using a calibrated densitometer (GS-800 Imaging Densitometer, Bio-Rad, Munich, Germany). The primary breast cancer protein lysate UPA497 was used as a positive control for DSC2, with its DSC2 expression being defined as 100% for the purpose of standardisation. Protein expression values in all detected bands were also normalised using the loading control β-Actin. For the statistical analyses, these values were divided into four equal groups (quartiles), representing very low, low-moderate, moderate and high protein expression.

### Breast cancer cell lines, cell culture and stable transfections

The human TNBC cell line MDA-MB231 and its brain seeking subline MDA-MB231-BR were provided by Dr Takara (University of Texas). Cells of both lines were cultivated in Dulbecco’s Modified Eagle’s Medium (DMEM, ThermoFisher Scientific, Waltham, MA, USA) supplemented with 10% fetal calf serum (FCS) under standard cell culture conditions. Cells were authenticated before usage. Two different DSC2-knockdown MDA-MB231-BR cell lines were generated by lentiviral transduction using vectors containing shRNA-sequences targeting specific regions of the DSC2 mRNA sequence (MISSION shRNA III and V, Sigma-Aldrich, GmbH). Similarly, a control cell line was established using a scramble shRNA sequence (Addgene, plasmid ID1864). The full DSC2 cDNA sequence obtained from a commercially available vector (Des476-Desmocollin 2-myc Plasmid; Addgene Plasmid ID: 32233) was cloned into LeGO-iC2-Puro + Plasmid (kindlykindly provided by AG Fehse, Center for Oncology, Department of Stem Cell Transplantation, UKE, Hamburg, Germany) using BamHI and EcoRI restriction enzymes. After lentiviral production in HEK293T cells, MDA-MB231 cells were transduced. The corresponding empty vector was taken as a negative control. After selection with puromycin (2ug/mL), the level of DSC2 mRNA and protein was detected using real time quantitative polymerase chain reaction RT-PCR and western blot analysis, respectively.

### RNA isolation and real-time quantitative polymerase chain reaction

RNA was isolated using the RNeasy Mini Kit (Qiagen, Hilden, Germany) and a QIAshredder (Qiagen, Hilden, Germany), and was subsequently reverse transcribed using qScriber cDNA Synthesis Kit (HighQu, Kraichtal, Germany) according to the manufacturer’s instructions. RT-PCR was carried out with ORA™ qPCR Green ROX H Mix (HighQu, Kraichtal, Germany) using the StepOnePlus System (Applied Biosystems, Thermo Fisher Scientific Inc.). The data analysis was performed using the ΔΔCt method. The following primers were used for DSC2: forward primer, 5’-GCCCATCTTCTTCTTGTCGTT-3’; reverse primer, 5’-CCCGTCTTGGTGAAAAAGTGT-3’. Primer sequences for the housekeeping gene were as follows: forward primer, 5’-GTCAGTGGTGGACCTGACCT- 3’; reverse primer, 5’ -TGCTGTAGCCAAATTCGTTG-3’.

### Immunofluorescence

Cells (1 × 10^5^) were seeded on coverslips, cultured for 48 h, and fixed with 3.7% formaldehyde for 20 min at room temperature. After blocking with 1% bovine serum albumin (BSA) in phosphate buffered saline (PBS) (Mg+/Ca+) for 1 h at room temperature, cells were incubated with a polyclonal DSC2 antibody (1:50 in 1% BSA/PBS; Sigma-Aldrich, Hamburg, Germany) over night at 4 °C. Subsequent to washing, cells were incubated with a second antibody solution (mouse anti-rabbit IgG Alexa Fluor® 488; Jackson ImmunoResearch, Ely, UK; 1:500 in 1% BSA/ PBS) for 1 h at room temperature. Coverslips were carefully placed on slides using mounting medium and DAPI (Vectashield). Images were acquired using a fluorescence microscope BZ9000 and the software BZII Viewer (Keyence, Germany).

### Proliferation assay

For cell proliferation analyses, the Cell Proliferation Kit II (XTT) (Roche Applied Science, Mannheim, Germany) was used according to the manufacturer’s instructions. Briefly, cells were seeded in a final volume of 100 µl medium per well in a 96-well plate (1.5 × 10^3^ MDA-MB231-BR cells per well, 2 × 10^3^ MDA-MB231 cells per well). After 24 h, 48 h and 72 h, cell viability was determined by adding XTT labelling mixture and by measuring the absorbance after 6 h at 490 nm using a microplate reader (DIAS Max002, Dynex Technologies, Chantilly, USA). Each experiment was performed with 12 replicates (wells) per condition (*n* = 12). Images shown are representative of three independently performed experiments.

### Cytotoxicity analysis

For the MDA-MB231-BR cell line and the DSC2-knock down sublines, an Annexin-V/PI staining was performed after cisplatin treatment in order to quantify the extent of apoptotic and necrotic cells. Briefly, cells were seeded into 6-well plates at a density of 2.5 × 10^5^ cells per well, incubated for 24 h and treated with cisplatin (Accord Healthcare Limited, North Harrow, United Kingdom) in three different concentrations (10 µM, 25 µM, 50 µM) for 48 h using serum-reduced DMEM medium. Subsequently, cells within the supernatant, as well as adherent cells, which were carefully detached using AccuMax (eBioscience, San Diego, CA, USA), were stained with an APC-labelled Annexin-V antibody (Annexin-V-APC, AnxA100, MabTag GmbH, Oldenburg, Germany) for 30 min at 4 °C in the dark. After washing with PBS (+/+), cells were resuspended in 1% BSA in PBS and stained with PI (BD Pharmingen, San Diego, CA, USA). FACS analysis was performed using the FACS Calibur (BD Biosciences, Heidelberg, Germany) and all data were analysed using FlowJo Software.

DSC2 overexpressing and control MDA-MB231 cells display a strong fluorescence, due to the transduction with the previously mentioned LeGO-iC2-Puro + Plasmid, which includes the mCherry-coding sequence. For these cell lines, the cisplatin-induced cytotoxicity was assessed using XTT, as described above. Briefly, MDA-MB231 cells were seeded into 96-well plates with 2 × 10^4^ cells in 100 µl per well. After incubating for 24 h, cells were treated with cisplatin in three different concentrations (10 µM, 25 µM, 50 µM) for 48 h and the Cell Proliferation Kit (Roche) was used as described in the previous section. Each experiment was performed in duplicates. Images shown are representative of three independently performed experiments.

### Migration assay

To investigate cell migration, the Oris™ Universal Cell Migration Kit (Platypus Technologies, Madison, WI) was used according to the manufacturer’s protocol. Briefly, cells were seeded (5 × 10^4^ cells in 200 µl per well) in a 96-well plate fitted with sterile silicon stoppers using serum-reduced medium (5% FCS). After 24 h incubation, stoppers were gently removed allowing cells to migrate into the central cell-free detection zone. Migration potential was assessed by analysing the cell-free area of each well at four different time points (0 h, 24 h, 48 h and 72 h post removal of stoppers) with the ImageJ Wound Healing Tool (Wayne Rasband, National Institute of Health). Each experiment was performed with 12 replicates (wells) per cell line. Images shown are representative of three independently performed experiments.

### Invasion assay

Matrigel Growth Factor Reduced (BD Biosciences, Heidelberg, Germany) was diluted to a concentration of 3.5 mg/ml with serum-free medium. Afterwards, 96-well plates were coated with a 1:1 mixture of Matrigel and serum-reduced medium (5% FCS) and incubated for 30 min at 37 °C. Hereafter, Oris™ Universal Cell Migration Kit (Platypus Technologies, Madison, WI) was used according to the manufacturer’s protocol as described above. After a 24 h incubation period, the stoppers and medium were carefully removed and 40 μl of newly prepared Matrigel coating solution was added. Plates were incubated again for 30 min at 37 °C. Finally, serum-reduced medium was added to all wells. For determining cell invasion potential, analyses were performed as described above. Each experiment was performed with 12 replicates (wells) per cell line. Images shown are representative of three independently performed experiments.

### Cell spheroid formation

Cells were seeded at 5 × 10^3^ cells in 200 μl medium per well on 2% agarose-coated (UltraPure™ Agarose, Invitrogen, Carlsbad, CA, USA, dissolved in PBS) 96-well plates. To assess and observe spheroid formation of cells, spheroids were examined and documented every second day using light microscopy and a camera (Axiovert 40 C, Carl Zeiss AG, Leica DFC320, Wetzlar, Germany). For further investigation of compactness, spheroids were dissociated by pipetting each spheroid up and down five times and by comparing formation immediately afterwards. Each cell line was seeded in quadruplicates. Images shown are representative of three independently performed experiments.

### Cytotoxicity in a 3D Model

Cisplatin-induced cell cytotoxicity on 3D-structures was assessed using immunocytochemical detection of phosphorylated gamma H2AX (γH2AX)—an established marker for DNA double-strand breaks—on tumour cell aggregates grown on polyHEMA (Sigma-Aldrich) coated flasks. Here, cell lines were seeded at a density of 2 × 10^6^ cells in 12 ml per polyHEMA coated T75 culture flask and cultured as cell aggregates for 72 h. Subsequently, cells were incubated with cisplatin in a final concentration of 50 μM for 8 h and 24 h, and were subsequently fixed in formalin, embedded into 2% agar (Agar NOBEL, Difco Laboratories, Detroit, MI, USA) and then embedded in paraffin. Slides from FFPE-cells were pre-treated in a steamer (citrate buffer pH 6.0) at 125 °C for 4 min and S1699 (pH 6.0, DAKO) at 121° C for 10 min, respectively) and incubated with an anti-DSC2 (1:25 HPA011911, Atlas Antibodies, Sigma-Aldrich, Hamburg, Germany) or an anti-γH2A.X antibody (1:10,000; ab81299, abcam, Berlin, Germany) for 1 h at room temperature. Incubation with biotin-labelled swine anti-rabbit secondary antibody occurred for 30 min at room temperature (1:200 dilution in TBS, E0353, DAKO, Glostrup, Denmark). For detection, sections were incubated with Vectastain® ABC-AP Kit (Vector Laboratories, Burlingame, CA USA) for 30 min and stained with Permanent Red (K0640, DAKO, Glostrup, Denmark). Rabbit immunoglobulin normal fraction (X0903, Agilent, Santa Clara, CA, USA) was used as a negative control for the anti-yH2A.x primary antibody. A rabbit polyclonal IgG (ab37415, abcam, Berlin, Germany) was used as a negative control for anti-DSC2 primary antibody. All slides were slightly counterstained with haematoxylin. Stained slides were scanned using the Axio Scan.Z1 (Zeiss, Jena, Germany) and images were acquired using netScope Viewer Pro software version 1.0.7079.25167 (NetBase Software GmbH, Freiburg, Germany). For quantifying the γH2A.X staining, manual counts of positive stained cells (4 × 100 cells in 4 different areas of each slide) were performed using the assistant electronic memory counter Counter AC-15 (Karl Hecht Assistant, Altnau; Switzerland).

### Intracardiac metastasis mouse model

The intracardiac mouse model was conducted as previously described [33]. Briefly, female 8 to 9-week-old SCID mice (CB17/lcr-Prkdcscid/lcrlcoCrl) were anesthetized and 1 × 10^6^ tumour cells were injected intracardially into the left ventricle of the heart (n = 15 per group). Tumour cells were previously transduced with luciferase-bearing plasmid and bioluminescence signals were tested before injection via bioluminescence imaging (BLI). After intracardiac injection, tumour cell dissemination was monitored weekly under BLI. Assessment of subsequent metastases was monitored in vivo weekly by imaging for up to 3 weeks. Mice showing termination criteria were immediately sacrificed. At the endpoint (21 days), animals were anesthetized and blood was collected from the left ventricle by cardiac puncture immediately before the final killing was executed by cervical dislocation. Ex vivo bioluminescence imaging was conducted from the lungs and brain. Lungs and brain were equally divided and frozen down for DNA isolation and subsequent ALU-PCR or paraffin-embedded for further analysis (H&E and luciferin staining) as previously described [33, 34]. The animal experiments were approved by the Authority for Social Affairs, Family, Health, and Consumer Protection of the Free and Hanseatic City of Hamburg through application N005/2020.

### Histology and immunohistochemistry

The whole brain and the right lung of the mice were fixed in 4% buffered formalin and processed for wax histology. 4 µm sections were cut from brain for immunohistochemistry and 10 sections from the middle of the block were stained with hematoxylin and eosin (H.E.). The lungs were fixed en block and subsequently cut into 1 mm thick slices and embedded in 2% agar. Afterwards, the lung slices were paraffin-embedded and cut into 4 µm thick sections. Ten sections of each paraffin wax block were H.E. stained and metastases were counted at a 200-fold magnification using Zeiss Axiophot photomicroscope (Zeiss, Jena, Germany). Additionally, two series of serial sections out of the middle of each paraffin wax block were preserved for further immunohistochemical analyses. The immunohistochemical staining was performed on 4 µm sections. Sections were deparaffinized in descending ethanol concentrations and pre-treated with citrate buffer solution (pH 6.1) in a steamer for 4 min. After incubation for 1 h at room temperature with the primary antibody DSC2 (Atlas, HPA011911), samples were washed twice with TBS-T (TBS + 0.1% TWEEN-20) and once with TBS for 5 min. After incubation with anti-rabbit secondary antibody (LS-Bio, LS-C350860) for 30 min at room temperature, antibody binding was visualized using the Vectastain ABC-AP Kit (VectorLabs., Burlingame, CA, USA) and Permanent Red Solution (Dako) according to the manufacturer’s instruction. The nuclei were counterstained in Mayer’s hemalum solution.

### Circulating tumour cell detection

Mouse blood samples (200–500 µl) were obtained via cardiac puncture and collected into EDTA KE/1.3 tubes (Sarstedt, Germany). To perform cardiac puncture, mice were deeply anaesthetized under isoflurane, and a 21-gauge needle coated with heparin was inserted into the heart. Mice were euthanized immediately following the cardiac puncture. Blood samples were processed on the label-independent, microfluidic system Parsortix® (ANGLE plc., United Kingdom), a device designed for the size-based capture of rare cells from whole blood [35]. The isolated cells were harvested and spun onto a glass slide (190 g, 7 min). Slides were dried overnight at room temperature and stored at − 80 °C until further analysis.

Tumour cells isolated with the Parsortix® system were identified via immunocytochemistry. Briefly, dried cytospin slides were brought to room temperature and fixed with 2% PFA (Sigma Aldrich, Germany) for 10 min. The samples were washed with 0.5 mL of 1x-PBS prior to permeabilization with 0.1% Triton X 100/PBS (Sigma Aldrich, Germany) for 10 min. Following two additional wash steps, 10% AB-serum/PBS (BioRad, Germany) was applied for blocking (60 min). Standard detection of CTCs is usually achieved with epithelial antibodies [36], however, TNBC cells lack epithelial markers and are successfully detected with CD298 [37]. Subsequently, directly anti-human PE labelled CD298 (clone LNH-94, Biolegend, USA) and anti-mouse Alexa Fluor 488 conjugated CD45 (clone HI30, Biolegend, USA) antibodies were incubated for 60 min, followed by 5 min of DAPI-incubation (1 µg/mL). Cytospins were covered with Prolong Gold Antifade Reagent (Thermo Fisher Scientific, Dreieich, Germany), sealed with a cover slip and examined by fluorescence microscopy (Axio Observer 7, Zeiss). CD298-positive, DAPI-positive, CD45-negative cells with intact morphology were defined as tumour cells. Clusters were defined when 2 or more cells were found together.

### Statistical analyses

All statistical analyses were performed using SPSS Statistics version 24 for Windows (IBM, Armonk, NY, USA). Correlations between mRNA and protein expression values were assessed using two-sided Pearson tests. Chi-square tests were used to correlate both mRNA expression (Microarray data, cohort A) and protein expression (WB data, cohort B) with the following clinical and pathological parameters; histological grading (G1/G2/G3), molecular subtype (Luminal/HER2 positive/TNBC), ER and PR Status (positive/negative) and the presence of metastases (loco-regional/bone/lung/visceral/brain). Kaplan–Meier estimates and the log-rank test were carried out to ascertain and compare disease-free and overall survival. The associated hazard ratios for the multivariate analyses were determined by Cox regression. Proliferation assays and cytotoxicity assays measured with XTT were statistically analysed using GraphPad Prism 5 (GraphPad, La Jolla, CA, USA). Each in vitro assay was performed at least three times. Statistical significance was assessed using unpaired two-tailed Student’s t-test. The assumption of homogeneity of variances was checked via Levene’s Test of Equality of Variances (p > 0.05). Results are given as mean ± s.d. or s.e. Probability values (*p*-value) ≤ 0.05 were considered to be statistically significant.

## Results

### High DSC2 mRNA levels are associated with the triple negative breast cancer subtype, an increased brain and lung metastasis risk and a decreased disease-free and overall survival for breast cancer patients

DSC2 mRNA levels were evaluated in 197 tumour samples of breast cancer patients using microarray data from our own cohort (see description in material and methods). Two different probe sets (204750_s_at and 204751_x_at) corresponding to DSC2, as well as the mean value of these two probe sets, were analysed. For each probe set and the mean value, the cohort was firstly divided into quartiles (Q) according to DSC2 expression: low (Q1), moderate-low (Q2), moderate-high (Q3), and high (Q4). Correlations between DSC2 mRNA levels and clinicopathological factors such as histological grading, stage, lymph node involvement, oestrogen and progesterone receptor status (ER, PR) and molecular subtype, showed a significant association between high DSC2 levels (Q4) and higher tumour grading (in one probe set), ER- (in one probe set and the mean value), PR-negativity (in one probe set) and molecular subtype (in two probe sets and the mean value), whereas no correlation between DSC2 and tumour stage or nodal status was found. Further, high DSC2 mRNA levels significantly correlated with the development of cerebral metastases (in one probe set and the mean value). Survival analyses showed a significant correlation between high DSC2 values and a shorter overall survival (in both probe sets and mean value) and disease-free survival (in both probe sets and the mean value). All mentioned statistical correlations using DSC2 quartiles or continuous values are listed in the Additional file 4: Table S3, including the corresponding p-values.

**Fig. 1:**
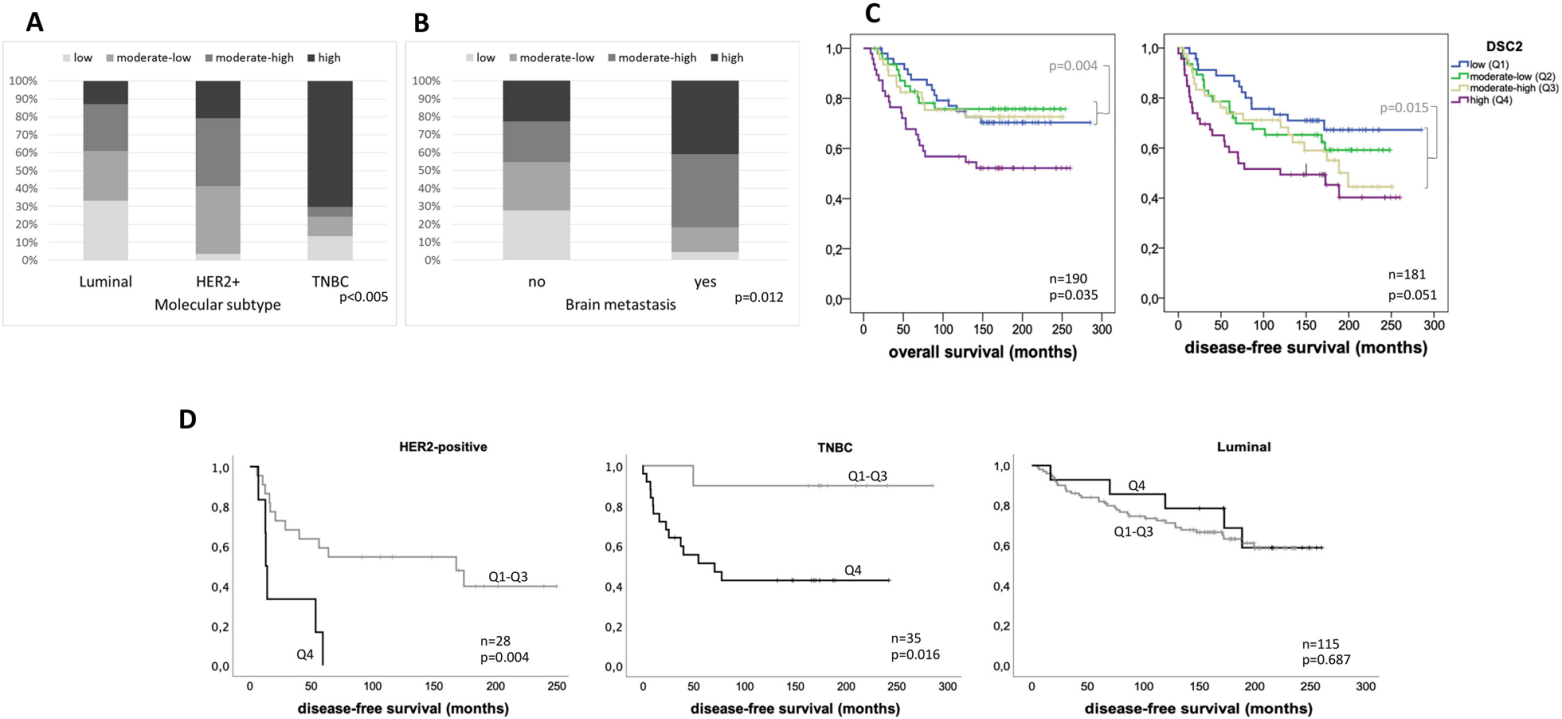
Quantitative expression of DSC2 mRNA in primary breast cancer tissue and its correlation with clinicopathological variables. **A** The TNBC molecular subtype is significantly associated with high DSC2 expression (p < 0.005). **B** A highly significant association between the development of cerebral metastases and high DSC2 expression was observed (p = 0.012). **C** Both, the disease-free survival (p = 0.051; right) and the overall survival (p = 0.035; left) are reduced in patients whose tumours express high levels of DSC2. **D** Stratification analysis showed a significant association between high DSC2 levels and shorter disease-free survival only in patients with HER2-positive and TNBC

For the DSC2 mean values, further analyses were carried out and are represented in Fig. 1. Here, HER2-positive and TNBC, which represent the most aggressive molecular subtypes, display higher DSC2 mRNA levels in comparison to the luminal subtype (p < 0.005; Fig. 1A). Also, primary tumours from patients that developed brain metastases showed higher DSC2 levels than those with no cerebral metastases (p = 0.012; Fig. 1B). Kaplan Meier analysis showed a significant association between high DSC2 levels and shorter overall survival using quartiles (p = 0.035; Fig. 1C) and a clear trend in the correlation between DSC2 and disease-free interval (p = 0.051; Fig. 1C). When we grouped patients included in quartiles Q1 to Q3 and compare them with patients with high DSC2 level (Q4) the differences in overall and disease-free survival became stronger (p = 0.004 and p = 0.015, respectively; Fig. 1C). In multivariate Cox regression analyses, including clinical stage, nodal involvement, and molecular subtype, DSC2 remained a prognostic indicator for disease-free survival (p = 0.008; HR 2.071; 95% CI 1207–3553, Additional file 4: Table S3). Interestingly, in a stratified survival analysis using this cut-off (cut-off-Q4), we found that the impact of DSC2 on overall survival (not shown) and disease-free survival was limited to the group of TNBC and HER2-positive patients (p = 0.0016 and p = 0.004, respectively; Fig. 1D), whereas in patients with luminal breast cancer the DSC2 level did not influence the survival time.

The impact of DSC2 on disease-free survival could be corroborated in a second cohort including 572 breast cancer samples (see description in material and methods and Additional file 1: Fig. S1). Here, we found a significant correlation of DSC2 mRNA expression with molecular subtype (p < 0.001; Additional file 1: Fig. S1A), brain and lung metastasis formation (p = 0.003 and p < 0.001, respectively; Additional file 1: Fig. S1B, C), as well as with disease-free survival (p < 0.001; Additional file 1: Fig. S1D) and, specifically, brain metastasis-relapse free survival (p < 0.001; Additional file 1: Fig. S1E).

### Western blot analyses detected a wide variance in DSC2 expression in primary breast cancers and a significant association with DSC2 mRNA levels

**Fig. 2:**
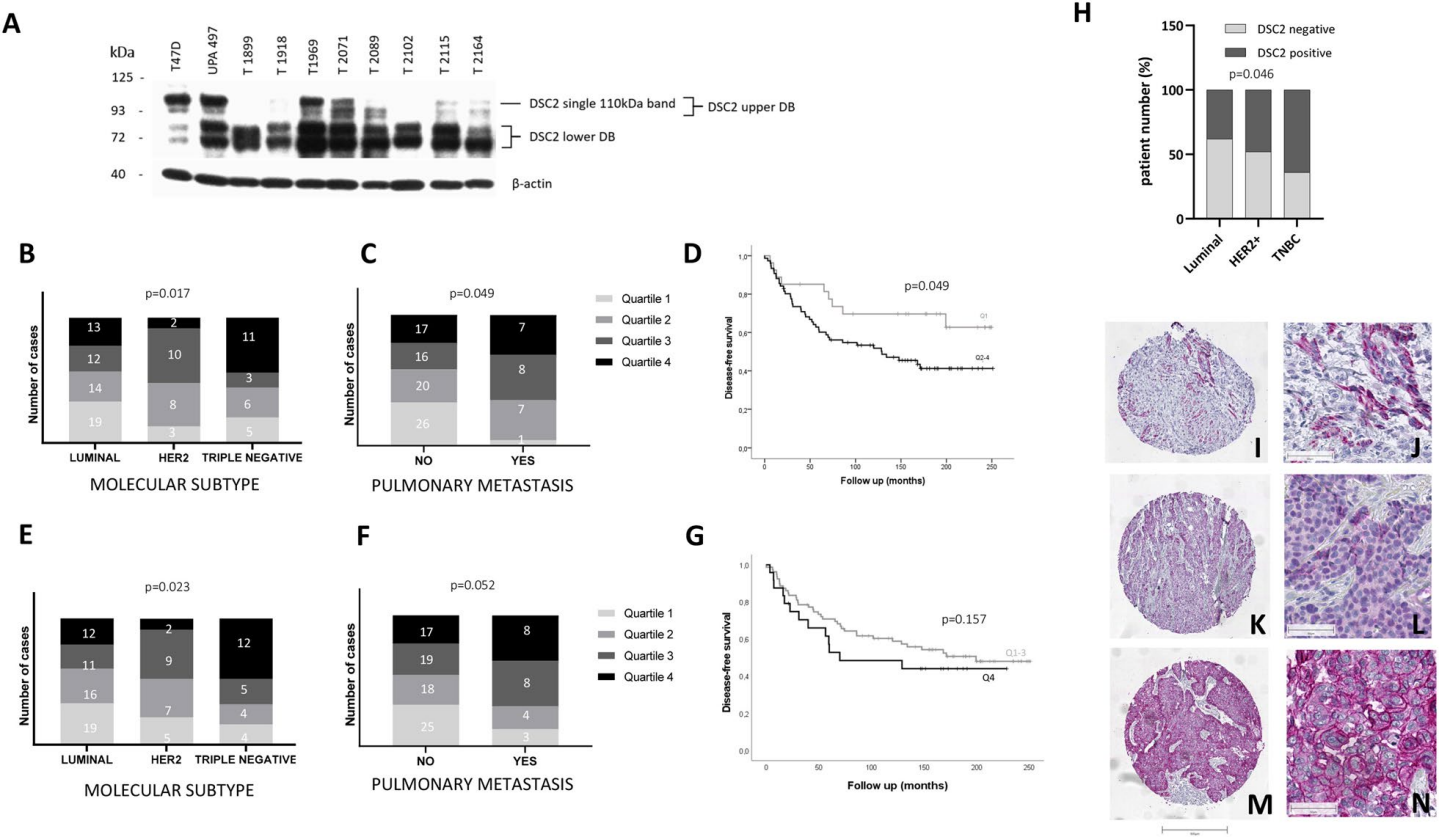
Quantitative expression of DSC2 in primary breast cancer protein lysates and its correlation with clinicopathological variables. **A** Western blots depicting representative expression of DSC2 in a variety of primary breast cancer samples. Protein lysates from the breast cancer cell line T47D, as well as the breast cancer lysate UPA 497, were used as positive controls for DSC2. β-actin protein expression was quantified as a loading control. Tumours with high DSC2 levels are significantly associated with the TNBC molecular subtype (**B** all WB DSC2 bands; p = 0.017 and **E** upper WB DSC2 band; p = 0.023). A significant association was observed between high DSC2 expression and the development of pulmonary metastases (**C** all WB DSC2 bands; p = 0.049 and **F** upper band; p = 0.052). Disease-free survival in accordance with DSC2 expression levels: Patients with a moderate to high DSC2 expression level have a significantly shorter disease-free survival, compared with patients whose tumours possess no or little DSC2 (**D** all WB DSC2 bands; p = 0.049 and **G** upper WB DSC2 band; p = 0.157). **H** Immunohistochemical analysis of primary breast cancer tissue confirmed a significant association between high DSC2 levels and TNBC subtype (p = 0.046). Representative DSC2 staining results from luminal (**I**–**J**), HER2 positive (**K**, **L**) and TNBC (**M**, **N**)

Of the 197 tumour samples tested in the microarray analyses, 111 were further analysed at a protein level using western blot. Patient characteristics were similar in both groups (Additional file 2: Table S1). The western blot results showed a wide range of DSC2 expression, varying between very low and very high expression. Additional to the expected band at 110 kDa, as described in the biological information provided by the antibody manufacturer, one or more additional bands were detected by the anti-DSC2 antibody in most samples (Fig. 2A). These bands presented, for the majority of the samples, as two double band sets between 60 and 85 kDa, and 93 and 110 kDa respectively. All samples were (at least lightly) positive for at least one of these double band sets. Since the origin and nature of these additional bands was not clear, both double band sets (individually and combined), as well as the single DSC2 band at 110 kDa (as specified by the manufacturer) were quantified by densitometry. The single band at 110 kDa and the double band between 93 and 110 kDa correlate with a coefficient of r = 0.936, while all bands combined (both double band sets) show a correlation of r = 0.676 (both p < 0.0000; Additional file 5: Table S4). In addition, all bands showed a moderate and significant correlation with DSC2 mRNA expression as obtained via microarray analyses (Pearson correlation coefficient r > 0.4; p < 0.001; Additional file 5: Table S4). These results suggest that all bands are isoforms and/or a result of different posttranslational modification, i.e. glycosylation. In the following analyses, we confine ourselves to the collective assessment of all detected DSC2 bands, as well as to the upper band, although similar results were obtained from analyses of each individual band, as well as each double band set.

The significant trend between molecular subtype and DSC2 expression seen at mRNA level could also be demonstrated at a protein level. While high DSC2 levels were significantly associated with the TNBC subtype, moderate and low DSC2 levels were more often observed in the HER2 and luminal subtypes respectively (p = 0.017 and p = 0.023; Fig. 2B and E). This association was further substantiated by the findings that tumours lacking PR- and ER- expression were likewise individually significantly associated with high DSC2 levels (all bands: p = 0.046 and p = 0.04 and upper band: p = 0.054 and p = 0.012 respectively; Additional file 1: Fig. S2). High DSC2 expression was, at the protein level, significantly associated with an increased risk of pulmonary metastases (all bands: p = 0.049 and upper band: p = 0.052; Fig. 2C and F). There was no significant association between high levels of DSC2 and increased risk of developing cerebral metastases in our cohort, although a trend towards this effect was observed (all bands: p = 0.405, upper band: p = 0.42). Patients whose tumours possessed increased levels of DSC2 sustained a shorter disease-free survival period in comparison to patients with low DSC2 expression (all bands: p = 0.049 and upper band: p = 0.157 Fig. 2D and G). No significant trend was observed between the patients’ overall survival and the level of DSC2 expressed in their tumour.

### Immunohistochemical verification of DSC2 expression in primary breast cancer samples

To further validate our findings at the protein level, DSC2 immunohistochemical staining was carried out on primary breast cancer samples included in four different tissue microarrays (TMAs). From the original 340 punctures included in the TMAs, only 243 could be evaluated. The remaining, non-analysable punctures were missing, lacking tumour cells or necrotic. Further, only primary breast cancer tissue was included in our analyses (n = 226), adjacent healthy tissue or metastatic tissue was excluded. DSC2 expression was observed exclusively in tumour cells in all biopsies. In positively stained tumour cells, we observed mainly a membranous staining, although DSC2 could be also detected in a minority of samples within the cytosol. Patient survival data was only available for a small group of samples and therefore corresponding correlation analyses were not performed. Information regarding the molecular subtype was available for 175 tissue samples. Here, we evaluated 21 luminal, 77 HER2 positive and 77 TNBC tissue samples. A positive DSC2 staining was detectable in 38% of the luminal, in 48% of HER2 positive and 64% of TNBC biopsies (Fig. 2H). While all positive stained luminal samples showed a moderate staining (2 < IRS < 6), approx. 20% of HER2 positive and TNBC tumours displayed a strong DSC2 staining (IRS > 6). Figure 2 shows representative DSC2 staining results from luminal (Fig. 2I, J), HER2 positive (Fig. 2K, L) and TNBC (Fig. 2M–L).

### Generation of DSC2-knockdown and overexpression in triple negative breast cancer cell lines

**Fig. 3:**
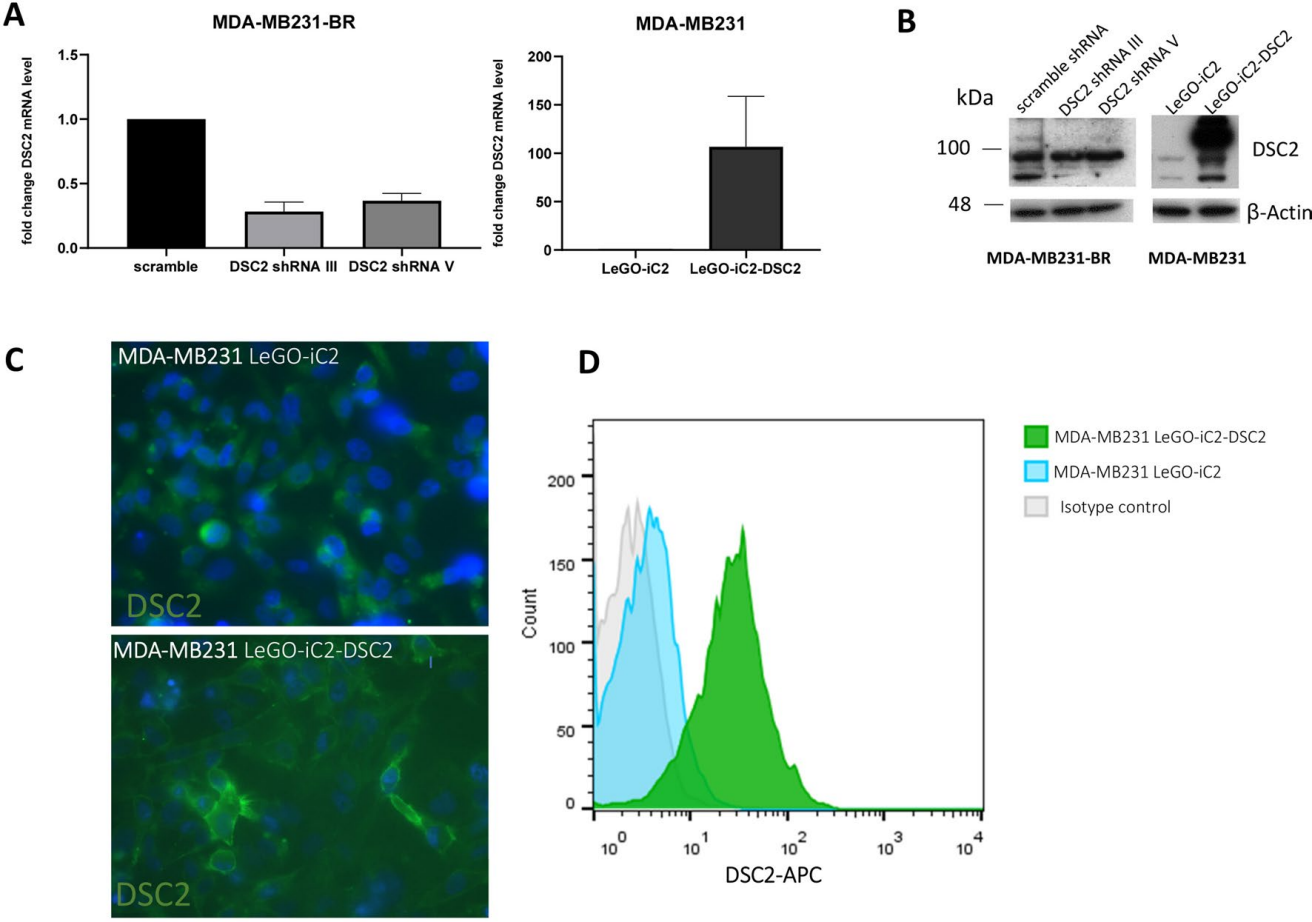
Desmocollin 2 knock down and overexpression in TNBC cell lines. DSC2 knock down in MDA-MB-231-BR cells and DSC2 overexpression in MDA-MB-231 cells were performed and validated at mRNA level via qRT-PCR (**A**) and protein level using Western Blot (**B**). Additionally, membranous DSC2 expression was visualized and quantified by immunofluorescence (**C**) and flow cytometry (**D**)

To assess the impact of DSC2 in breast cancer cells, we performed DSC2 knockdown in MDA-MB231-BR cells, which endogenously express a higher level of DSC2 than the parental cell line MDA-MB231. Among all sublines generated by transfection with 5 different DSC2-specific shRNAs sequences, shRNA III and V—which showed around 70% and 60% DSC2-knockdown at mRNA level (Fig. 3A) respectively, compared to the scramble control—were chosen for further analysis. A clear reduction in the DSC2 expression level in these cells could be corroborated by western blot (Fig. 3B). In addition, we chose the parental cell line MDA-MB231 for DSC2 overexpression. Here, stable lentiviral transduction with the LeGO-iC2 DSC2 vector induced a strong DSC2 overexpression in comparison with the empty vector-transduced cells, as detected by qRT-PCR and western blot (Fig. 3B). Here, the membranous localisation of DSC2 was further corroborated using immunofluorescence and flow cytometry (Fig. 3C and D, respectively).

### Influence of DSC2 expression on the proliferation, migration, invasion and chemosensitivity of breast cancer cells in a 2D cell culture

**Fig. 4:**
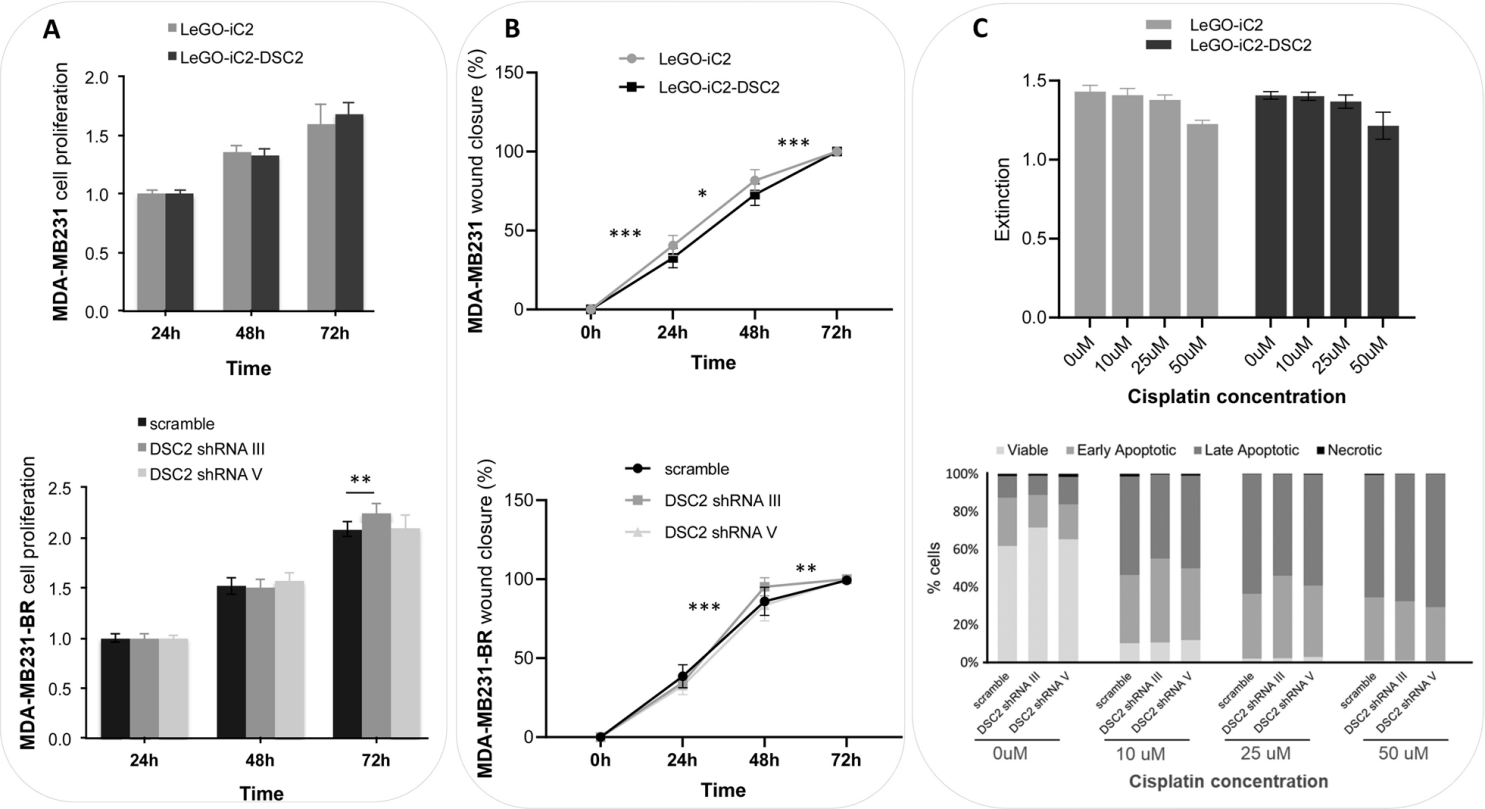
Impact of DSC2 knock down and DSC2 overexpression on tumour cell proliferation, migration and chemosensitivity in 2D. **A** DSC2 overexpression in MDA-MB231 cells does not affect cell proliferation (upper panel). A slight increase in the proliferation rate was detected in one of the DSC2 knock out clones and only after 72 h cultivation (lower panel). **B** DSC2 overexpression in MDA-MB231 and DSC2 silencing in MDA-MB231-BR cells slightly but significantly decreased (upper panel) and increased (lower panel) the tumour cell migratory ability in a wound closure assay, respectively. **C** Both DSC2 overexpression (upper panel) and silencing (lower panel) did not show any impact on tumour cell chemosensitivity against cisplatin

The impact of reduced or increased DSC2 expression on diverse cellular properties of the TNBC cells was analysed in 2D cell culture conditions using the stably transfected MDA-MB231-BR and MDA-MB231 cells as previously described. We observed no significant differences in cell proliferation between MDA-MB231 control cells and those with increased DSC2 expression (LeGO-iC2 DSC2; Fig. 4A, upper panel). A significant increase in proliferation was only observed between MDA-MB231-BR scramble control cells and those with reduced DSC2 expression (only in shRNA III cells) after 72 h. As this result was observed only in 1 of 3 experiments (Fig. 4a, lower panel), we conclude that a DSC2 down-regulation does not affect cell proliferation in the brain-seeking cell line. In wound healing assays with silicon stoppers, DSC2 knock down in the shRNA III clone led to a significant (yet slight) increase in cell migration and therefore to an accelerated wound closure, whereas the shRNA V clone resembled the scramble control. Accordingly, DSC2 overexpression decreased cell migration and therefore slowed wound closure, however the difference is again too small to be conclusive (Fig. 4B).

Furthermore, when comparing MDA-MB231-BR cells with reduced DSC2 expression to MDA-MB231-BR control cells, no significant differences in the amount of apoptotic and necrotic cells, measured by flow cytometry and Annexin V and PI staining, respectively, could be noted after exposure to the cytostatic drug cisplatin for 48 h. The chemosensitivity of MDA-MB231 cells was also not affected after DSC2 overexpression, as measured using an XTT-assay (Fig. 4C).

### Impact of DSC2 expression on breast cancer cell aggregation and chemosensitivity in a 3D cell culture

**Fig. 5:**
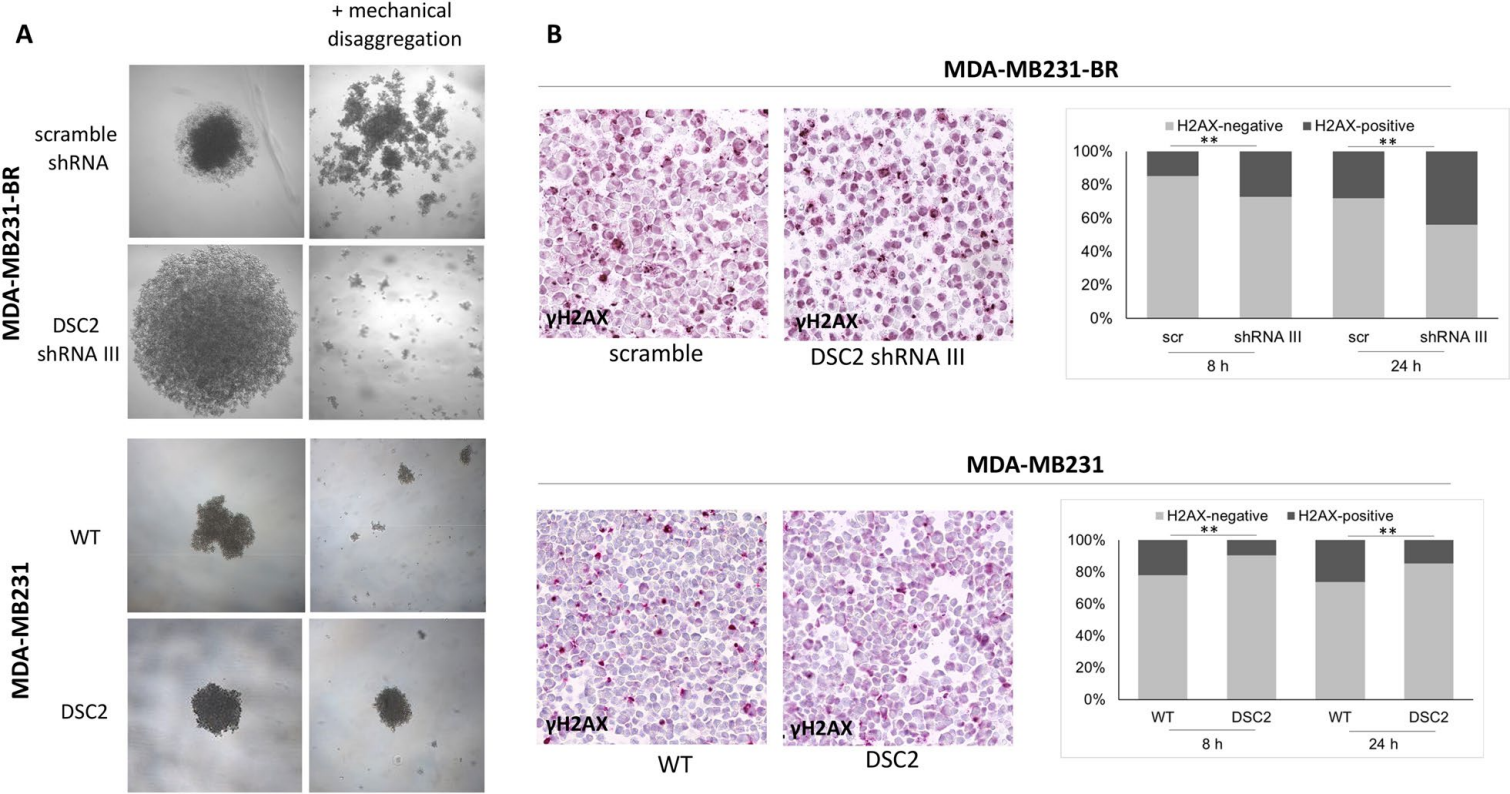
Impact of DSC2 knock down and DSC2 overexpression on tumour cell aggregation and chemosensitivity in 3D. **A** DSC2 knock down in MDA-MB231-BR cells led to an impaired tumour cell aggregation in a 3D spheroid formation assay (upper panel), whereas DSC2 overexpression increased the compactness of the spheroids in comparison to WT, which could not be dissociated by mechanical disaggregation. **B** DNA double-strand breaks were quantified in MDA-MB231-BR DSC2 knock down cell vs. control (upper panel) and in MDA-MB231 DSC2 overexpressing cells vs. WT growing in 3D (polyHEMA) after cisplatin treatment using immunocytochemical detection of phosphorylated H2AX (γH2AX). The quantification of the aforementioned staining revealed a significant increase of cisplatin-induced cell cytotoxicity in DSC2 knock down cells (upper panel) and a significant reduced amount of DNA double-strand breaks in DSC2 overexpressing cells (lower panel)

We next analysed the impact of DSC2 overexpression and DSC2 knock down on the cellular properties of the two TNBC cell lines in a 3D model. We observed clear differences in the aggregation capacity between the different sublines, as shown in Fig. 5A. Here, MDA-MB231-BR cells with reduced DSC2 expression (shRNA III) failed to build compact spheroids in comparison to the corresponding control cells (scramble shRNA), when seeded on agarose coated surfaces. The differences in the aggregation capacity and compactness were even more clear after mechanical disaggregation by slowly pipetting, showing full disaggregation mostly to single cells and small cell aggregates of only a few cells in the DSC2 knock down cells, whereas the control cells remained as small cell aggregates that were able to adhere to each other again over time. Contrary to our expectations, MDA-MB231 cells with enhanced DSC2 expression did not show any phenotypical differences in the spheroid formation assay in comparison to the corresponding empty vector control cells. However, we observed a clearly enhanced compactness—which could be confirmed after mechanical disaggregation—when compared with the wild type parental cell line (WT). Quantification of the DSC2 mRNA level in the parental cell line revealed a DSC2 up-regulation (f.c. 2.3; Additional file 1: Fig. S3) in the control cell line (LeG-iC2) compared with WT cells. This unspecific and treatment-induced DSC2 deregulation in the MDA-MB231 LeGO-iC2 cells might explain the unexpectedly similar behaviour of the two sublines in the 3D model.

In order to study whether the mechanical stiffness of the cellular aggregates further influences drug diffusion and, in turn, chemosensitivity, we cultured cells of the aforementioned cell lines with different DSC2 expression levels on polyHEMA coated culture flasks, allowing them to form cell aggregates in suspension, and treated them subsequently with cisplatin for 8 and 24 h. The differences in spheroid shape observed in the aforementioned agarose model were also observed under polyHEMA culture conditions. Quantification of cisplatin-mediated DNA intra-strand cross-links (Additional file 1: Fig. S4 & materials and methods) and cisplatin-induced cell cytotoxicity on 3D cellular structures were assessed using immunocytochemical detection of PT-GG adducts and phosphorylated gamma H2AX (γH2AX)—an established marker for DNA double-strand breaks-, respectively. Here, we observed a significant increase in the number of platin adducts and DNA damaged cells in the MDA-MB231-BR subline with reduced DSC2 expression (shRNA III) compared with the control cells (scramble). Correspondingly, we observed a significantly reduced number of platin adducts and γH2AX positive cells in the MDA-MB231 cell line with DSC2 overexpression, when compared with the WT (Fig. 5B).

### Reduced DSC2 expression in MDA-MB-231-BR cells decreases metastatic potential in vivo

**Fig. 6:**
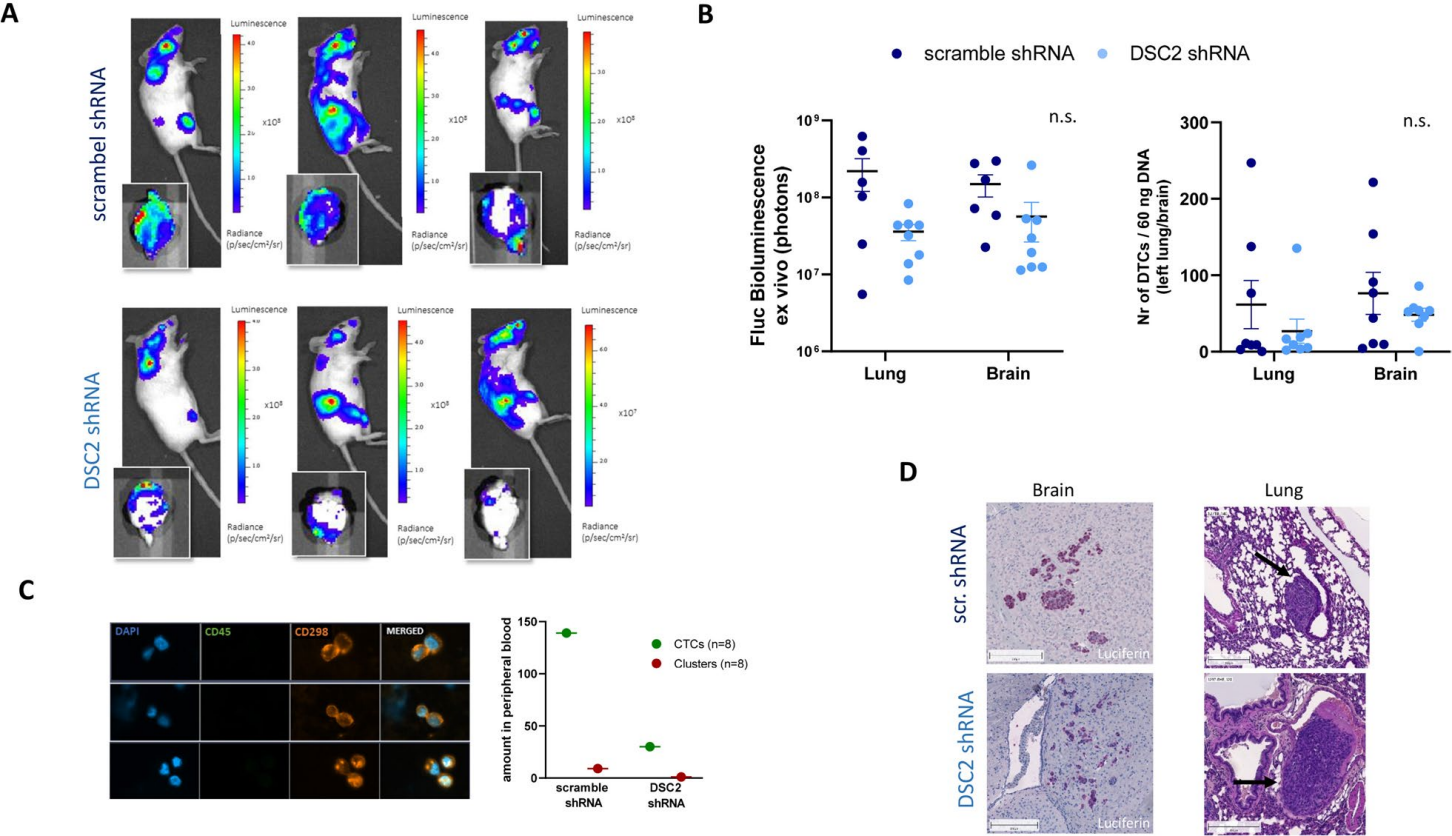
Role of DSC2 on TNBC cell dissemination in an intracardiac mouse model in vivo*.* **A** Representative BLI pictures of whole mice and the corresponding ex vivo brain of each group (MDA-MB231-BR DSC2 shRNA and MDA-MB231-BR scramble shRNA) 21 days after injection. **B** The quantification of ex vivo BLI signals (*left panel*) and amount of disseminated tumour cells (ALU-PCR; *right panel*) from brains and lungs revealed a lower metastastic load in organs from mice bearing DSC2 knock down tumours in comparison with those injected with the control cell line. **C** Reduced levels of CTCs and CTCCs (circulating tumour cell clusters) were quantified in the DSC2 knock down group (n = 8, blood was pooled for analysis) in comparison to the control group (n = 8, blood was pooled for analysis). Representative pictures of CTCCs isolated from the DSC2 knock down group showing CD298 positivity and a CD45 negative staining. **D** Small metastatic lesions in the brain and lungs were detected by specific luciferase staining. In the brain of DSC2 knock down mice mainly single cells or small cell aggregates were detected, whereas larger metastases were found in the brains of the control scramble mice group. Microscopically detectable intravasal lung metastases in a DSC2 knock down mice and established metastatic lesion in a control mouse. Black file marks metastatic tumour cells

Next, the role of DSC2 in TNBC cells during metastasis development was analysed in vivo. Here, using the brain-seeking cell line MDA-MB-231-BR, the corresponding scramble and DSC2 shRNA cells (shRNA III) were injected (1 × 10^6^ cells each) intracardially into the left ventricle of 8–9 weeks old female SCID mice. Although 15 mice per group were injected initially, 12 mice showed poor physical condition or strong bioluminescence signals in the lungs and thus needed to be removed at the beginning of the experiment. Therefore, 9 mice in the control group and 10 mice in the DSC2 shRNA group were further monitored weekly by BLI. One mouse from the control group and two from the DSC2 shRNA group showing hind limb paralysis were sacrificed on day 18. The remaining mice were sacrificed 21 days after cancer cell injection. Figure 6A shows representative bioluminescence images of three mice and their corresponding ex vivo brains from each test group 21 days after injection. The overall tumour burden in both mice groups did not strongly differ, while ex vivo bioluminescence signals in the scramble vs. DSC2 shRNA group (mean values: brain = 2,2 × 10^8^ vs. 3,6 × 10^7^ and lung = 1,5 × 10^8^ vs. 3,4 × 10^7^ photons/sec), as well as the amount of disseminated tumour cells (DTCs) in the brain and lung by ALU-PCR (mean values: brain = 76,5 vs. 48,3 and lung = 61,6 vs. 23,9 DTCs/60 ng DNA) clearly showed a reduced metastatic load in the DSC2 shRNA group (Fig. 6A and D). In line with this finding, we found a reduced number of circulating tumour cells (CTCs) and circulating tumour cell clusters in the blood samples from mice corresponding to the DSC2 knock down group compared to the control group (Fig. 6C). The histological analysis of the brains and the tumour cell visualization using a specific luciferase staining (Fig. 6D) showed indeed smaller metastatic lesions in DSC2 knock down mice, consisting mostly of single cells or small cell aggregates, whereas larger metastases were found in the brains of the control mice group. The presence of microscopically detectable lung metastases was also confirmed upon histological examination of the right lung. In the DSC2 knock down mice, we detected more intravascular metastases than established pulmonary metastatic lesions, when compared to the control group (Fig. 6D).

## Discussion

Desmosomes are important structures for intercellular adhesion and are functionally present most abundantly in tissue exposed to high levels of mechanical stress, such as the epidermis and myocardium [38]. In recent years, desmosomal proteins have become a point of interest in cancer research. Depending on protein type and primary tumour localisation, both tumour-enhancing and tumour-suppressive effects of desmosomal protein up/down-regulation have been observed [39]. Desmoglein 2, desmocollin 2 (DSC2) and plakophilin 1 (PKP1) have been recently linked to an increased metastatic potential of breast cancer cells by promoting cell clustering and enhanced survival during the tumour cell dissemination process [25, 31]. In the present study, we have analysed the role of DSC2 as a prognostic and predictive factor for primary breast cancer and the development of breast cancer metastases. In order to address this question, DSC2 levels at mRNA and protein levels were correlated with clinical and histopathological data. Our research has been able to show, for the first time, that higher levels of DSC2 in primary breast cancer tissue significantly influence disease progression and metastatic behaviour in HER2 positive and TNBC patients. Functionally, the extent of DSC2 expression in tumour cells directly impacts their capacity to aggregate and, in turn, influences their chemosensitivity, as shown in in vitro analyses after DSC2 up-regulation and DSC2 silencing in the TNBC cell line MDA-MB-231 and its brain-seeking subline MDA-MB-231-BR, respectively. In vivo DSC2 knock down reduces the amount of circulating tumour cells and clusters, and consequently the amount and size of established brain metastases and established metastatic lesions in lung tissue.

As mentioned, desmosomal proteins and specifically DSC2 have been previously investigated in the context of cancer research. For example, in colorectal cancer, DSC2 loss enhances tumour cell growth by altering the Akt/β-catenin signal pathway [29]. Furthermore, knockdown of desmosomal proteins such as DSC2, DSG2 and plakoglobin was reported to impair cell aggregation and reduce anoikis resistance in lung and breast cancer cells, while their expression levels were correlated with poor overall survival in lung cancer patients and poor metastasis-free survival in breast cancer patients [19]. A similar effect of DSC2 and DSG2 mediated cell adhesion on cell aggregation was detected in colon cancer spheroids [20]. Aceto et al. detected an up-regulation of plakoglobin and other desmosomal components in CTC clusters of breast cancer patients and revealed its importance for the formation of CTC clusters and distant metastases [9]. And, in a more recent study, breast and lung cancer cells resistant to shear stress revealed an up-regulation of DSC2 and PKP1, leading to more CTC cluster formation and enhanced cell survival in circulation via activation of the PI3K/AKT/Bcl-2 pathway [31]. Here, our findings suggest that the aforementioned DSC2 mediated effect on tumour cell aggregation and survival applies for TNBC as well.

In the present study, we found that tumour DSC2 levels significantly influence the disease-free and overall survival of breast cancer patients, in particular for patients with primary tumours corresponding to the HER2 positive and TNBC molecular subgroups. Two independent microarray datasets and a western blot cohort corroborated the unfavourable prognostic role of DSC2 and, together with immunohistochemical analysis on an independent cohort, demonstrated a significantly increased expression of the desmosomal protein in the most aggressive molecular subtypes, namely the aforementioned HER2 positive and TNBC. Our results have further highlighted the potential of DSC2 as a predisposing marker for the development of breast cancer metastases to the brain and lungs, and are in line with a previous work by Landemaine et al. who identified DSC2 as a potential predictive marker for lung metastasis in breast cancer [40]. With the rising incidence of breast cancer cerebral metastases, and the lingering difficulty in treating the disease, the ability to identify high risk patients who would benefit from increased prevention would be of great clinical value. In line with a previous study on brain metastasis samples from patients with different tumour entities, which identified DSC2 as a potential marker for brain metastasis development [27], we found that high DSC2 mRNA expression significantly correlated with an increased risk for cerebral and lung metastases, although for the first localization, this trend could not be validated at a protein level. High DSC2 protein expression was, however, significantly associated with the development of pulmonary metastases [41].

Our findings challenge the self-evident hypothesis that up-regulation of desmosomal proteins leads to a more mechanically cohesive primary tumour, and therefore a less aggressive one. Indeed, a possible explanation for the contrary findings is that enhanced tumour cell aggregation through DSC2 up-regulation is a factor which favours the development of CTC clusters, which have a considerably higher metastatic potency than singular CTC [9, 18]. In various studies, apoptotic morphology was detected in single CTCs, but not within CTC clusters. This finding supports the theory that clustering of CTCs leads to a higher metastatic potential by, for example, increasing the probability of survival in the circulation [11, 42]. Marrella et al. showed that shear stress affects CTC survival in vitro, with CTC clusters being more resistant to shear forces than single CTCs [43]. Increasing shear stress values incrementally caused disaggregation of CTC clusters. Shear stress resistant cells were found to express more DSC2 and DSC1 [31], and were more likely to form clusters. Additionally, a link has been drawn between increased CTC cluster density and size, and increased cluster cell survival in vitro [44]*.* Additional to an increased resilience, the formation of clusters—in particular those with a very high density—may lead to reduced chemosensitivity and, thus, a survival advantage compared to single CTCs [12, 44, 45]. Enhanced cell–cell interactions in CTC clusters could also confer resistance to anoikis, a form of apoptosis due to deprivation of cell–cell and cell–matrix contacts [12, 19]. Furthermore, heterogenous cluster formation with immune cells, such as macrophages or leucocytes, may provide a mechanism for immune escape [42]. Collectively, these findings indicate that DSC2 mediated cell adhesion is probably of greater functional importance in later steps of the metastatic cascade, such as survival within the circulation and chemoresistance, rather than in the process of dissolution of future metastatic cells from the primary tumour mass.

In the present study, up-regulation of DSC2 in breast cancer cells led, as expected, to an enhanced cellular aggregation capacity and thus the formation of tight 3D cell clusters, while tumour cell aggregates after DSC2 silencing displayed a looser structure which rapidly dissociated when subjected to mechanical stress. Interestingly, higher DSC2 expressing aggregates showed lower apoptotic rates than the corresponding control clusters when treated with cisplatin and, correspondingly, reduced DSC2 expression significantly enhanced tumour cell response to cisplatin. In contrast, we did not observe any effect of DSC2 up- or down-regulation on the chemosensitivity of both TNBC cell lines cultured as a monolayer, indicating that the DSC2-mediated cohesiveness of the 3D tumour cell clusters is the main reason for the altered chemosensivity. This finding highlights the relevance of in vitro 3D culture models to accurately mimic the in vivo conditions [46]. Tumour cell aggregation significantly influences cell response to cytotoxic drugs, as cells in a spheroid environment are more resistant to radiation and chemotherapeutic agents, a phenomenon known as multicellular resistance (MCR) that has been described for different anticancer drugs, including cisplatin [47]. Possible mechanisms of MCR include signalling-mediated inhibition of apoptosis, an increased proportion of quiescent cells, as well as reduced permeability and, in turn, impeded drug diffusion. Li et al. recently described high DSC2 and PKP1 levels in shear stress-resistant breast and lung cancer, which facilitate cell cluster formation and also activate the PI3K/AKT/Bcl-2–mediated pathway, thereby increasing cell survival [31]. However, the exact mechanism behind the observed DSC2-mediated chemoresistance in our model remains unclear and needs to be elucidated in the context of an ongoing project.

Under 2D culture conditions, up- or down-regulation of DSC2 was found to have only a minor effect on cell migration and invasion, although the tendency of our results is in line with recent studies on adhesion proteins (for example DSG2) and cell migration [25, 48]. No severe impact of increased or decreased DSC2 expression on cell morphology, proliferation or apoptosis could be detected in our 2D cell culture. These findings contrast with diverse reports describing a clear effect of DSC2 down-regulation on 2D proliferation and/or apoptosis in, for example, breast, prostate or oesophageal squamous cell carcinoma in vitro [28, 31, 49], with both a pro- and anti-tumorigenic role being postulated. Thus, the role of DSC2 seems to be entity-specific, or even subtype-specific, as shown in our study with a clear negative prognostic value of DSC2 in HER2 positive and TNBC, yet no impact on survival for patients with luminal breast cancer.

The results of the in vivo metastatic model, even though the size of the experiment did not allow a significant conclusion, clearly underline our hypothesis. Reduced DSC2 tumour expression decreases the amount of viable tumour cells in the blood circulation—CTCs as well as CTC clusters—and, as a consequence, reduces the effective formation of distant metastases. Our results are in line with those recently published by Li et al. showing the relevance of dual expression of DSC2 and PKP1 for cluster formation and survival in circulation in a lung cancer cell line in a tail vein injection model [31].

## Conclusions

Our results link high DSC2 expression with the TNBC molecular subtype, a higher breast cancer tumour grade and a significantly shorter disease-free survival. Further, we have been able to highlight the potential predictive value of DSC2 for the development of cerebral and pulmonary metastases. Functionally, DSC2 promotes tumour cell aggregation and, in turn, fosters the formation of tight CTC clusters with high metastatic potential. An interesting possibility for future research would be to expand the DSC2 analyses to circulating tumour cells and clusters, as well as pulmonary breast cancer metastases, in order to further clarify the potential of DSC2 as a breast cancer brain and pulmonary metastases marker for clinical use.

## Supplementary Information

**Additional file 1.** Supplementary Figures, Material and Methods.

**Additional file 2: Table S1.** Patient characteristics Microarray and Western Blot cohorts.

**Additional file 3: Table S2:** Patient characteristics TMAs.

**Additional file 4: Table S3:** Correlation between DSC2 mRNA levels and clinical and histopathological parameter.

**Additional file 5. Table S4:** Correlation between DSC2 mRNA level and protein level.

## Data Availability

The datasets used and/or analysed during the current study are available from the corresponding author on reasonable request.

## Abbreviations

CTCs: Circulating tumour cells
BBB: Blood–brain barrier
BCBM: Breast cancer brain metastases
BM: Brain metastases
DSC2: Desmocollin-2
SDS: Sodium dodecyl sulphate
WB: Western blot
SI: Stain intensity
PP: Percentage of positive cells
TMA: Tissue microarray
TBST: Tris buffered saline with tween
ABC-complex: Avidin–biotin enzyme complex
DAB: Diaminobenzidin
TBS: Tris-buffered saline
SPSS: Statistical Package for Social Sciences
TNBC: Triple negative breast cancer(s)
