# Supplementary material for: The role of the desmosomal protein desmocollin 2 in tumour progression in triple negative breast cancer patients
Source: Cancer Cell Int. 2023 Mar 16;23:47. doi: 10.1186/s12935-023-02896-9 (PMC10018948; doi:10.1186/s12935-023-02896-9)
Supplement: Supplementary file 1 — Additional file 1. Supplementary Figures, Material and Methods. [file 12935_2023_2896_MOESM1_ESM.docx]

**Additional file**

**Figure S1**. DSC2 Microarray Analysis in an independent validation cohort. For validation purpose we used an independent Affymetrix microarray dataset consisting of 572 breast cancer samples from Gene Expression Omnibus (GSE2603, GSE2034, GSE12276) for which detailed information on metastatic localization was available [PMID 19421193].


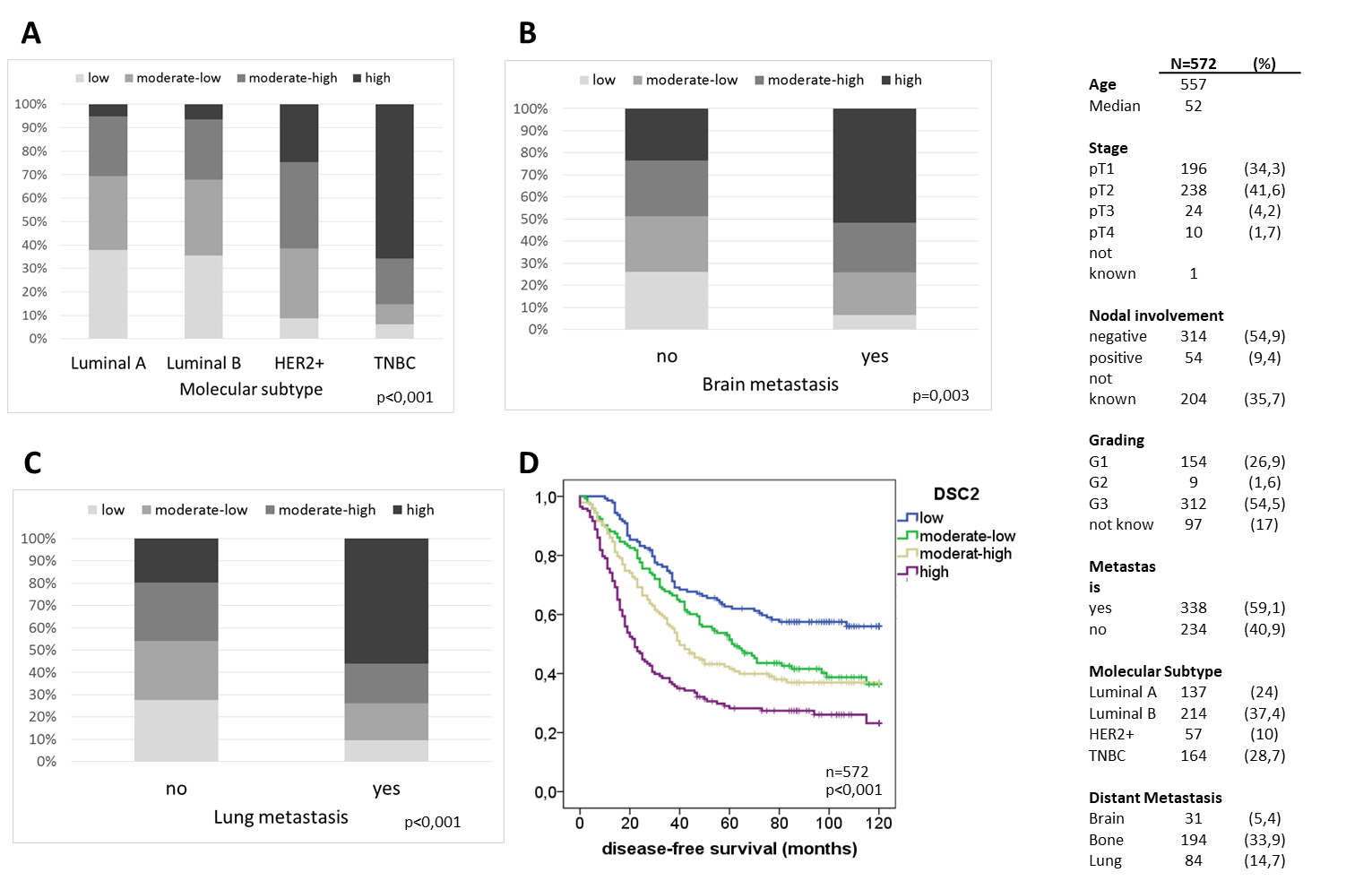


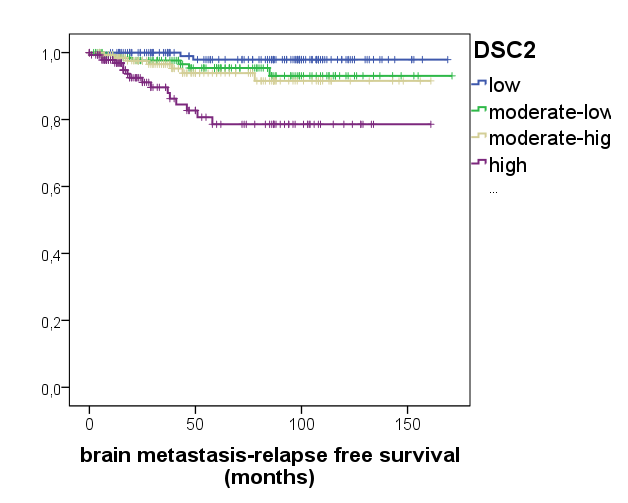


n=572

p<0,001

**E**

**Figure S2**. In Western Blot analysis PR- and ER- negativity were significantly associated with high DSC2 protein levels (all bands: left side and upper band: right side)

p=0.04


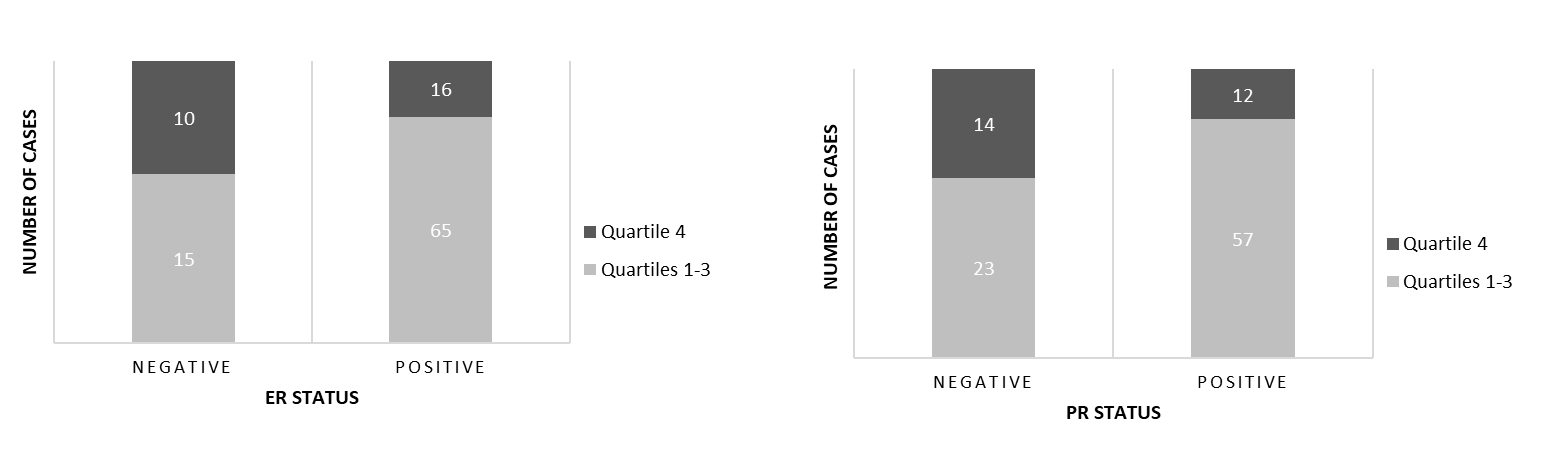


p=0.012


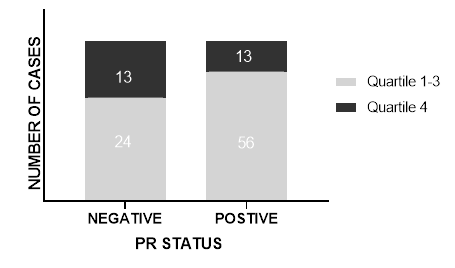

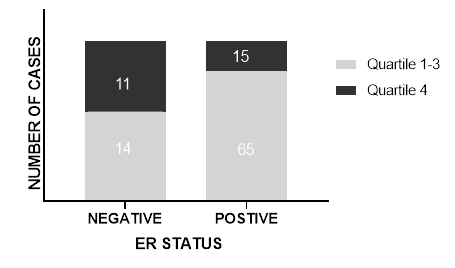


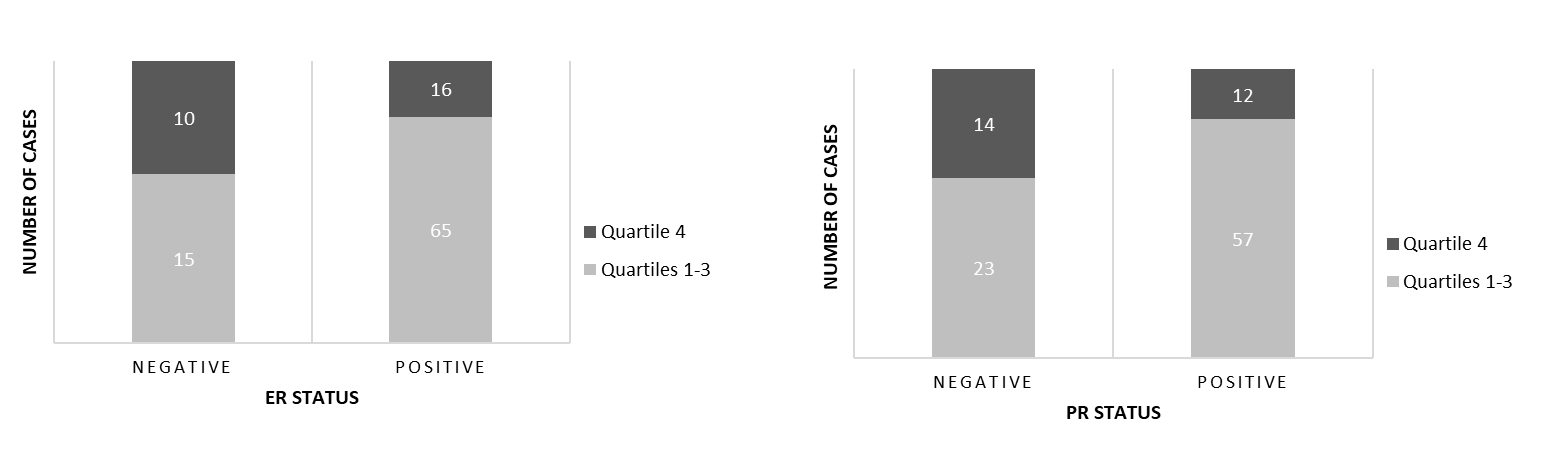


p=0.046

p=0.054

**Figure S3**. Overexpression of DSC2. (**A**) mRNA level in wild type (WT) MDA-MB-231-BR and after lentiviral lentiviral transduction with LEGO-iC2-DSC2 and the empty vector (LeGO-iC2). (**B**) Morphology of cell spheroids with MDA-MB-231-BR WT, LeGO-iC2 (empty vector) and LeGO-iC2-DSC2 (DSC2 overexpression) cells, before and after mechanical dissociation.

**
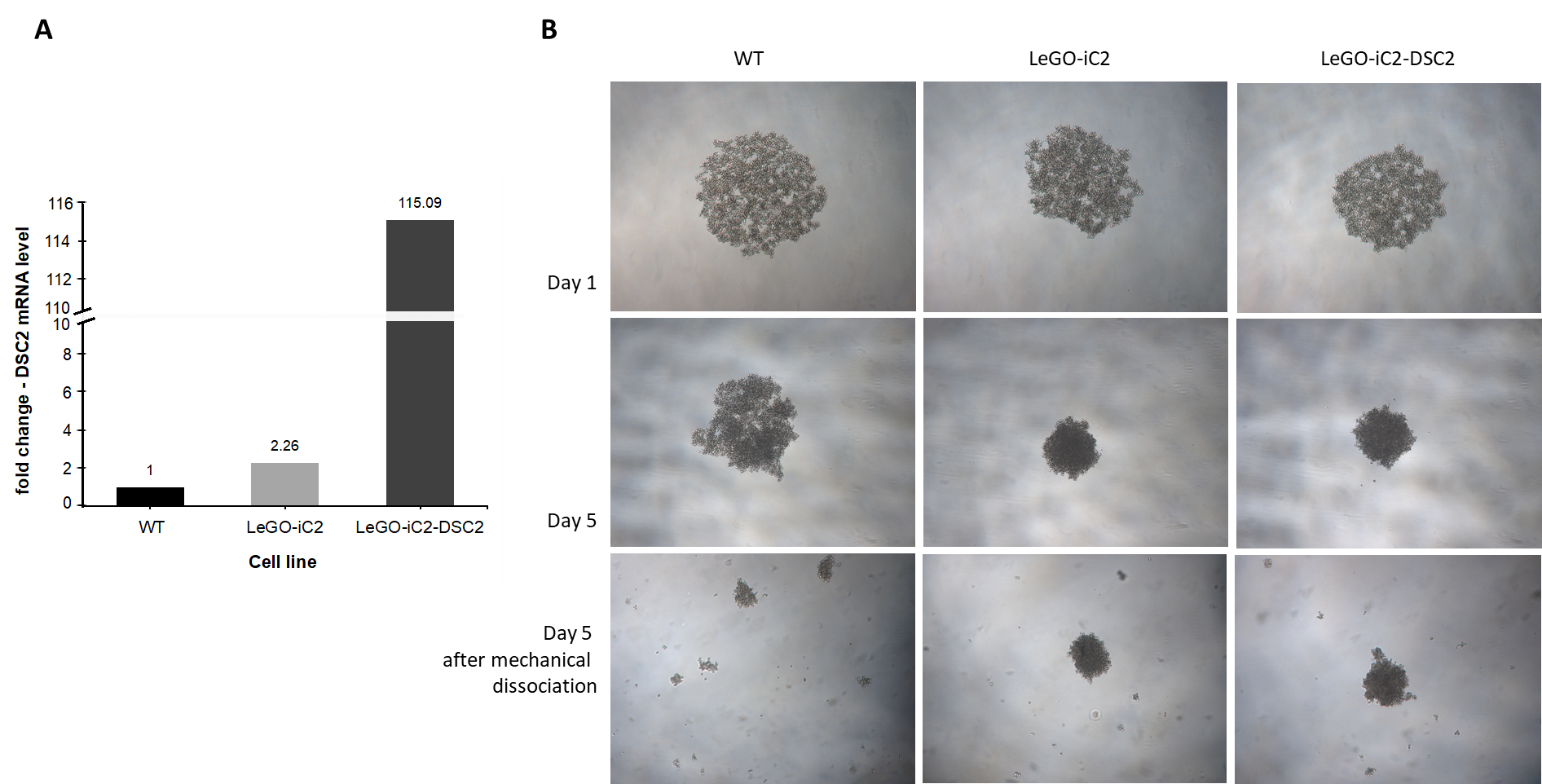
**

**Figure S4**. Cisplatin PT-GG staining. **(A)** Exemplary images of immunohistochemical PT-GG staining in MDA-MB231-BR scramble and DSC2 shRNA III after 8h treatment with cisplatin **(B)** Quantification of PT-GG positive and negative cells in MDA-MB231-BR scramble and DSC2 shRNA III after 8h and after 24h treatment with cisplatin **(C)** Exemplary images of immunohistochemical PT-GG staining in MDA-MB231-BR WT, LeGO-iC2 (empty vector) and LeGO-iC2-DSC2 (DSC2 overexpression) after 8h treatment with cisplatin **(D)** Quantification of PT-GG positive and negative cells in MDA-MB231-BR WT, LeGO-iC2 and LeGO-iC2-DSC2 after 8h and after 24h treatment with cisplatin.


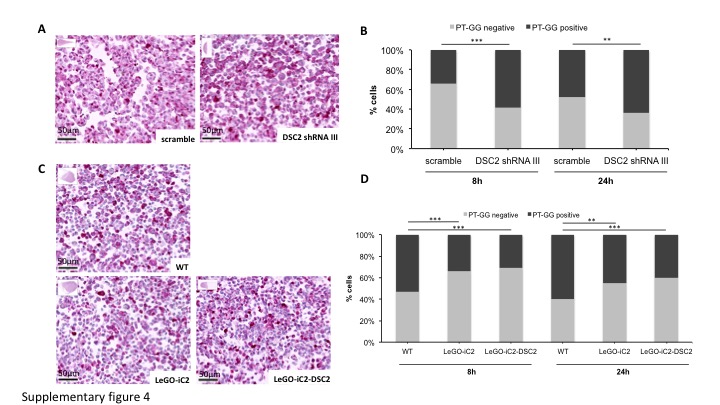


**Supplementary Material and Methods**

For staining of cisplatin DNA-adducts, slides were deparaffanized, pretreated with FastEnzyme (ZUC059-015, Zytomed, Berlin, Germany) for 5 min and incubated with 1:1000 diluted rat monoclonal PT-GG antibody (Oncolyze, Essen, Germany) for 80 min, respectively at room temperature. After washing, incubation with biotin-conjugated rabbit anti-rat antibody (1:100 dilution in TBS; 312-065-048, JacksonImmuno Research, Ely, UK) followed for 30 min at room temperature. Staining was visualized with streptavidin conjugated Alkaline Phosphatase and Chromogen RED using DAKO Real^TM^ detection System (K5005, DAKO, Glostrup, Denmark) kit according to the manufacturer’s instructions. As negative control, rat IgG 2a kappa Isotype Control (14-4321-85, eBioscience, San Diego, CA, USA) was used instead of primary antibody. All slides were counterstained with haematoxylin.
